# Epigenome-wide association study of serum cotinine in current smokers reveals novel genetically driven loci

**DOI:** 10.1186/s13148-018-0606-9

**Published:** 2019-01-05

**Authors:** Richa Gupta, Jenny van Dongen, Yu Fu, Abdel Abdellaoui, Rachel F. Tyndale, Vidya Velagapudi, Dorret I. Boomsma, Tellervo Korhonen, Jaakko Kaprio, Anu Loukola, Miina Ollikainen

**Affiliations:** 1 Institute for Molecular Medicine Finland (FIMM), University of Helsinki, Helsinki, Finland; 2 Department of Biological Psychology, Vrije Universiteit Amsterdam, Amsterdam, Netherlands; 3 Campbell Family Mental Health Research Institute, Centre for Addiction and Mental Health, and Departments of Pharmacology & Toxicology and Psychiatry, University of Toronto, Toronto, Canada; 4 Department of Public Health, University of Helsinki, Helsinki, Finland; 5 Department of Pathology, University of Helsinki, Helsinki, Finland

**Keywords:** Epigenome-wide association study, Smoking, Cotinine, Genetic risk score, Nicotine metabolism, meQTL, Causal inference, Molecular mediation

## Abstract

**Background:**

DNA methylation alteration extensively associates with smoking and is a plausible link between smoking and adverse health. We examined the association between epigenome-wide DNA methylation and serum cotinine levels as a proxy of nicotine exposure and smoking quantity, assessed the role of SNPs in these associations, and evaluated molecular mediation by methylation in a sample of biochemically verified current smokers (*N* = 310).

**Results:**

DNA methylation at 50 CpG sites was associated (FDR < 0.05) with cotinine levels, 17 of which are novel associations. As cotinine levels are influenced not only by nicotine intake but also by *CYP2A6*-mediated nicotine metabolism rate, we performed secondary analyses adjusting for genetic risk score of nicotine metabolism rate and identified five additional novel associations. We further assessed the potential role of genetic variants in the detected association between methylation and cotinine levels observing 124 *cis* and 3898 *trans* methylation quantitative trait loci (meQTLs). Nineteen of these SNPs were also associated with cotinine levels (FDR < 0.05). Further, at seven CpG sites, we observed a trend (*P < 0.05*) that altered DNA methylation mediates the effect of SNPs on nicotine exposure rather than a direct consequence of smoking. Finally, we performed replication of our findings in two independent cohorts of biochemically verified smokers (*N* = 450 and *N* = 79).

**Conclusions:**

Using cotinine, a biomarker of nicotine exposure, we replicated and extended identification of novel epigenetic associations in smoking-related genes. We also demonstrated that DNA methylation in some of the identified loci is driven by the underlying genotype and may mediate the causal effect of genotype on cotinine levels.

**Electronic supplementary material:**

The online version of this article (10.1186/s13148-018-0606-9) contains supplementary material, which is available to authorized users.

## Background

Smoking remains a major preventable cause of morbidity and mortality worldwide and has been shown to associate extensively with DNA methylation changes across the genome as evidenced by several epigenome-wide association studies (EWAS) [1]. Almost all EWAS so far, including a recent large meta-analysis (*N* = 15,907) [2], assessed the association between DNA methylation and self-reported smoking status or smoking quantity [3–23]. Self-reported smoking status and quantity, however, are prone to inaccuracies due to reporting bias (usually under reporting or recall bias) [24]. Cotinine, the primary metabolite of nicotine, is a reliable measure of nicotine exposure among current smokers [25] and provides higher statistical power compared to self-reported smoking quantity, as shown by Ware et al. [26] in a genome-wide association study (GWAS) meta-analysis of cotinine levels.

The cotinine GWAS meta-analysis [26] identified a locus on chromosome 4, within UDP glucuronosyltransferase family 2 member B10 (*UGT2B10*) gene, a key enzyme in nicotine and cotinine metabolism [27]. However, genetic variants in this gene did not associate with self-reported smoking quantity suggesting that cotinine may capture more than mere nicotine exposure [26]. Cotinine level is not only dependent on nicotine intake, but is also affected by the rate of formation from nicotine and the rate of cotinine metabolism to 3-hydroxycotinine, both are mediated by one highly genetically polymorphic enzyme, *CYP2A6* [28]. The rate of nicotine metabolism also influences smoking behavior; for instance, fast metabolizers tend to smoke more cigarettes per day and more intensely than slow metabolizers, likely due to the longer retention of nicotine among slow metabolizers from a given intake [29, 30]. Nicotine metabolite ratio (NMR; 3-hydroxycotinine/cotinine) is a reliable proxy for *CYP2A6*-mediated nicotine and cotinine metabolism [29]. NMR is highly heritable with just three independent SNPs accounting for ~ 30% of variance in NMR in Finnish population [31].

In this study, we performed an EWAS to examine the association of serum cotinine levels with DNA methylation in a sample of current (daily) smokers from the Finnish Twin Cohort (*N* = 310). To reduce the impact of variation in cotinine levels due to *CYP2A6*-mediated metabolism, we utilized the genetic risk score (GRS) of NMR. As many of the genes identified in our EWAS had genetic variants that were linked to smoking-related traits previously, we further investigated the effects of genetic variants on methylation (methylation quantitative trait loci (meQTLs)) and cotinine levels. Finally, at loci where the genetic variants were associated with both methylation and cotinine levels, we examined whether altered methylation maybe a molecular mediator in the association observed between the genotype (single nucleotide polymorphism; SNP) and cotinine levels rather than being altered directly as a consequence of smoking.

## Results

**Fig. 1:**
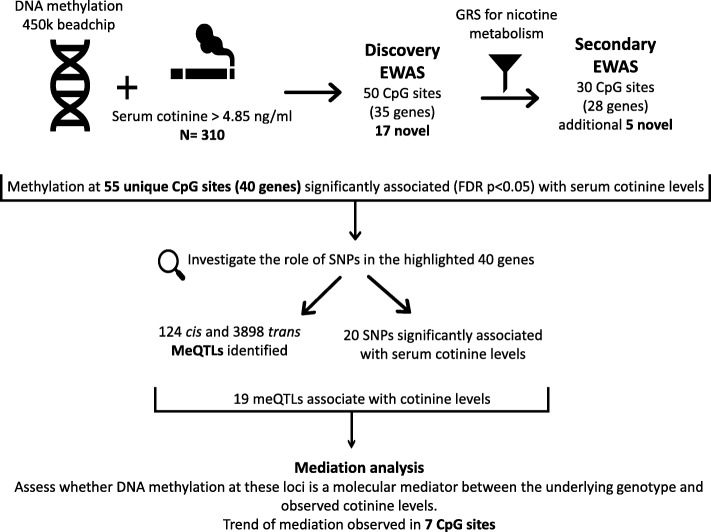
Study design and summary of results

The overall study design and summary of our main findings is shown in Fig. 1.

### Epigenome-wide association study

**Fig. 2:**
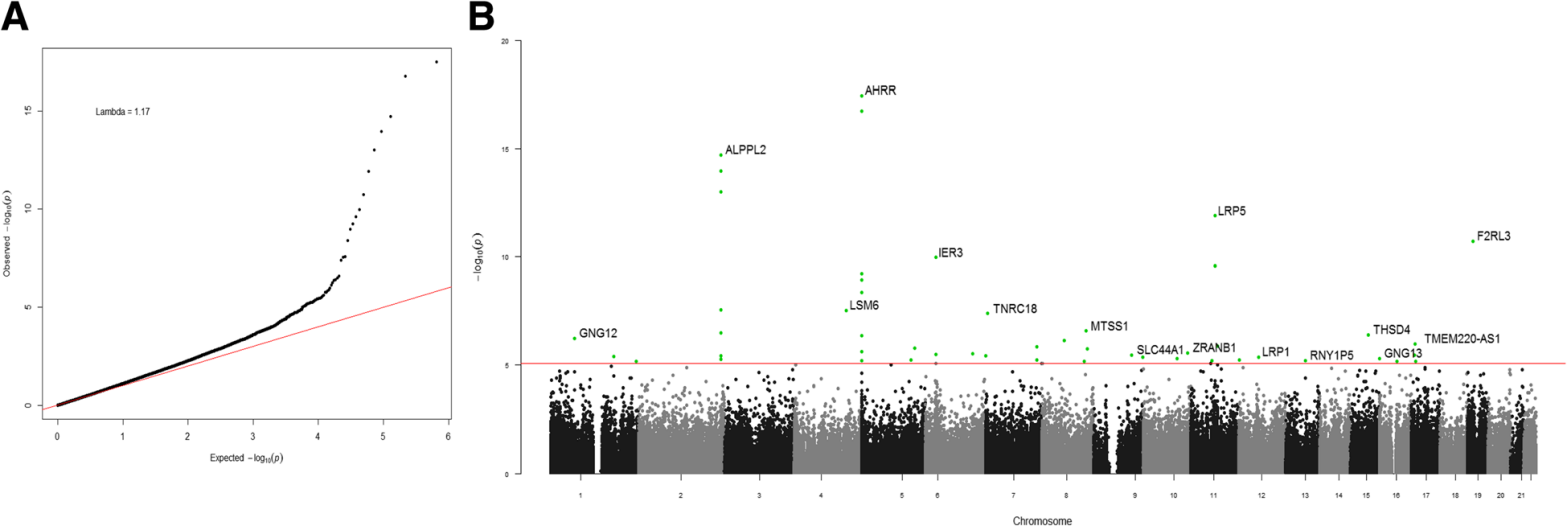
Manhattan and QQ plots showing epigenome-wide associations from discovery analysis. **a** QQ plot showing observed versus expected − log_10_(*P*) for association at all loci. **b** Manhattan plot showing chromosomal locations of − log_10_(*P*) for association at each locus. All CpG sites with FDR < 0.05 are highlighted in green and the top gene for each of the highlighted loci is labeled

We examined the association of nicotine exposure, assessed by serum cotinine levels, with DNA methylation in a sample of current smokers (*N* = 310, serum cotinine > 4.85 ng/ml). In our discovery EWAS, we identified 50 CpG sites (in 35 genes) showing significant association (FDR < 0.05) with serum cotinine levels (Table 1). Among the 50 highlighted CpG sites, 17 (in 17 genes) were novel associations while 33 (in 18 genes) have previously been reported to associate with smoking in EWAS. Consistent with previous studies, cg05575921 in *AHRR* was the most significant CpG site (FDR = 1.1 × 10^− 12^), with seven additional previously reported CpG associations identified within *AHRR*. Other notable replicated hits include six CpG sites in *ALPPL2* (min FDR = 2.0 × 10^− 10^ for cg05951221) and cg03636183 in *F2RL3* (FDR = 8.7 × 10^− 07^). For a great majority (84%, 42/50) of the highlighted CpG sites, higher cotinine levels were associated with lower methylation with small effect sizes. For instance, a 100 ng/ml increase of cotinine was associated with approximately 3.6% lower methylation at cg05575921 in *AHRR*. Novel CpG associations in smoking-related genes include cg13740236 in *LSM6* (FDR = 6.7 × 10^− 04^) and cg26589665 in *THSD4* (FDR = 6.9 × 10^− 03^) (full list in Table 1). Manhattan and quantile-quantile (QQ) plots for the discovery analysis are presented in Fig. 2.

**Table 1:** Results from the discovery and secondary epigenome-wide association analyses of serum cotinine levels in current smokers

CpG	Chr	Nearest gene	Discovery analysis				Secondary analysis				References	
			Beta	SE	*P* value	*P*_FDR_	Beta	SE	*P* value	*P*_FDR_		
cg25189904	1	*GNG12*	− 0.000135	0.000026	5.6E−07	9.0E−03	− 0.000134	0.000028	1.8E−06	3.1E−02	[2–13, 52]	
cg21033965	1	*CLEC20A*	− 0.000096	0.000020	4.0E−06	3.7E−02			**		[2]	
cg26687670	1	*OPN3*	− 0.000025	0.000005	6.5E−06	4.4E−02			**		Novel	
cg27241845	2	*ALPP*	− 0.000083	0.000016	3.3E−07	6.2E−03	− 0.000082	0.000016	8.8E−07	1.7E−02	[2–10]	
cg03329539	2	*ALPPL2*	− 0.000070	0.000012	2.7E−08	6.6E−04	− 0.000069	0.000013	1.3E−07	3.0E−03	[2–10, 12, 52]	
cg06644428	2	*ALPPL2*	− 0.000054	0.000012	5.5E−06	4.3E−02			**		[2–11, 19, 52]	
cg05951221	2	*ALPPL2*	− 0.000178	0.000021	1.9E−15	2.0E−10	− 0.000171	0.000022	1.4E−13	1.5E−08	[2–13, 19, 20, 52]	
cg21566642	2	*ALPPL2*	− 0.000210	0.000026	1.1E−14	8.8E−10	− 0.000205	0.000027	4.2E−13	3.4E−08	[2–12, 19, 20, 52]	
cg01940273	2	*ALPPL2*	− 0.000134	0.000017	9.8E−14	6.3E−09	− 0.000129	0.000018	3.4E−12	2.2E−07	[2–13, 20, 52]	
cg13193840	2	*ALPPL2*	− 0.000045	0.000010	3.6E−06	3.4E−02	− 0.000048	0.000010	2.5E−06	3.3E−02	[2–7, 9, 10]	
cg02306995	3	*SEMA5B*	*				0.000047	0.000010	4.4E−06	4.8E−02	Novel ***	
cg04776445	4	*MSX1*	*				0.000085	0.000018	4.3E−06	4.8E−02	Novel ***	
cg13740236	4	*LSM6*	− 0.000053	0.000009	2.9E−08	6.7E−04	− 0.000057	0.000010	1.0E−08	3.0E−04	Novel	
cg11902777	5	*AHRR*	− 0.000071	0.000011	1.1E−09	3.2E−05	− 0.000068	0.000012	2.1E−08	5.6E−04	[3–7, 9]	
cg01899089	5	*AHRR*	− 0.000063	0.000014	6.1E−06	4.4E−02	**				[2–8]	
cg05575921	5	*AHRR*	− 0.000359	0.000039	3.3E−18	1.1E−12	− 0.000351	0.000041	4.4E−16	1.1E−10	[2–13, 16, 18–20, 51, 52]	
cg26703534	5	*AHRR*	− 0.000086	0.000013	5.8E−10	1.9E−05	− 0.000084	0.000014	6.2E−09	2.0E−04	[2–8, 12, 18, 51, 52]	
cg14817490	5	*AHRR*	− 0.000099	0.000021	2.4E−06	2.9E−02	**				[2–10, 12, 13, 18, 52]	
cg25648203	5	*AHRR*	− 0.000091	0.000015	4.3E−09	1.1E−04	− 0.000086	0.000016	1.1E−07	2.8E−03	[2–13, 52]	
cg21161138	5	*AHRR*	− 0.000142	0.000016	1.8E−17	2.9E−12	− 0.000141	0.000017	6.6E−16	1.1E−10	[2–12, 18, 51, 52]	
cg24090911	5	*AHRR*	− 0.000076	0.000015	4.3E−07	7.4E−03	− 0.000074	0.000015	2.5E−06	3.3E−02	[2–7, 10]	
cg10961758	5	*EDIL3*	*				− 0.000069	0.000014	2.5E−06	3.3E−02	Novel***	
cg16179182	5	*VTRNA1–1*	0.000048	0.000010	5.7E−06	4.3E−02	**				Novel	
cg14580211	5	*SMIM3*	− 0.000062	0.000013	1.7E−06	2.2E−02	**				[2–9, 52]	
cg06126421	6	*IER3*	− 0.000132	0.000020	1.1E−10	4.3E−06	− 0.000132	0.000021	6.9E−10	2.8E−05	[2–12, 20, 52]	
cg14753356	6	*IER3*	− 0.000072	0.000015	3.1E−06	3.4E−02	**				[2–9]	
cg22856972	6	*TBPL1*	0.000066	0.000014	3.0E−06	3.4E−02	0.000073	0.000014	9.1E−07	1.7E−02	Novel	
cg05469934	7	*PDGFA*	0.000038	0.000008	3.6E−06	3.4E−02	**				Novel	
cg09022230	7	*TNRC18*	− 0.000079	0.000014	4.1E−08	8.8E−04	− 0.000070	0.000014	2.4E−06	3.3E−02	[2–6, 8]	
cg21322436	7	*CNTNAP2*	− 0.000055	0.000012	5.6E−06	4.3E−02	**				[2–7, 9]	
cg25949550	7	*CNTNAP2*	− 0.000045	0.000009	1.4E−06	1.9E−02	**				[2–9, 11, 52]	
cg09267815	8	*YTHDF3*	− 0.000080	0.000016	7.2E−07	1.1E−02	**				Novel	
cg22288066	8	*NOV*	0.000033	0.000007	6.8E−06	4.4E−02	**				Novel	
cg24838345	8	*MTSS1*	− 0.000088	0.000017	2.6E−07	5.3E−03	− 0.000084	0.000017	2.3E−06	3.3E−02	[2–6, 8, 9]	
cg25305703	8	*CASC8*	− 0.000088	0.000018	1.7E−06	2.2E−02	**				[2–7]	
cg01692968	9	*SLC44A1*	− 0.000075	0.000016	3.3E−06	3.4E−02	− 0.000079	0.000017	2.7E−06	3.3E−02	[2–9]	
cg14120703	9	*NOTCH1*	− 0.000043	0.000009	4.3E−06	3.8E−02	− 0.000045	0.000010	4.3E−06	4.8E−02	[2, 4]	
cg05992400	10	*CYP2C18*	− 0.000050	0.000011	4.9E−06	4.1E−02	**				Novel	
cg16421726	10	*ZRANB1*	0.000056	0.000012	2.7E−06	3.1E−02	**				Novel	
cg05335388	11	*ABTB2*	*				0.000035	0.000007	1.6E−06	2.9E−02	Novel***	
cg19254163	11	*PTGDR2*	− 0.000042	0.000009	6.4E−06	4.4E−02	**				[2–7]	
cg21611682	11	*LRP5*	− 0.000071	0.000010	1.2E−12	6.7E−08	− 0.000067	0.000010	8.2E−11	3.8E−06	[2–11, 52]	
cg14624207	11	*LRP5*	− 0.000063	0.000010	2.6E−10	9.2E−06	− 0.000063	0.000010	1.4E−09	5.1E−05	[2–7, 9]	
cg10788371	11	*LRRC32*	− 0.000064	0.000013	1.3E−06	1.8E−02	− 0.000067	0.000013	8.6E−07	1.7E−02	[2–6]	
cg11491407	12	*CACNA2D4*	− 0.000095	0.000020	5.7E−06	4.3E−02	**				Novel	
cg18443392	12	*LRP1*	0.000027	0.000006	4.2E−06	3.8E−02	**				Novel	
cg04524475	13	*RNY1P5*	− 0.000053	0.000012	6.2E−06	4.4E−02	**				Novel	
cg26589665	15	*THSD4*	− 0.000042	0.000008	3.8E−07	6.9E−03	− 0.000041	0.000008	2.1E−06	3.3E−02	Novel	
cg05361530	16	*GNG13*	0.000044	0.000009	4.8E−06	4.1E−02	**				Novel	
cg07991586	16	*ADCY7*	− 0.000042	0.000009	6.6E−06	4.4E−02	**				Novel	
cg05460226	17	*PIK3R5*	− 0.000072	0.000015	3.4E−06	3.4E−02	**				[2–4]	
cg05396243	17	*TMEM220-AS1*	− 0.000036	0.000007	1.0E−06	1.5E−02	**				Novel	
cg07658936	17	*ARHGAP44*	0.000029	0.000006	6.8E−06	4.4E−02	**				Novel	
cg03636183	19	*F2RL3*	− 0.000151	0.000022	1.9E−11	8.7E−07	− 0.000155	0.000022	2.8E−11	1.5E−06	[2–15, 17, 18, 20–22, 52]	
cg12817959	22	*SSTR3*	*				0.000034	0.000007	4.3E−06	4.8E−02	Novel***	

*CpG not genome-wide significant in discovery analysis

**CpG not genome-wide significant in secondary analysis

***Novel hits identified in secondary analysis

As cotinine levels are influenced not only by nicotine intake but also by nicotine and cotinine clearance rate (both mediated by *CYP2A6*), we performed a secondary analysis, wherein we included the GRS for NMR as an additional covariate in the model. In this secondary EWAS, altogether 30 CpG sites (in 20 genes) were significantly (FDR < 0.05) associated with cotinine levels. Five (in five genes) of these 30 CpG sites were novel and not genome-wide significant in the discovery EWAS (cg04776445 in *MSX1*, cg05335388 in *ABTB2*, cg10961758 in *EDIL3*, cg12817959 in *SSTR3*, and cg02306995 in *SEMA5B*), and the remaining 25 CpG sites (in 15 genes) overlapped with the discovery results. Half of the discovery EWAS hits (25 CpG sites) were no longer significant after controlling for nicotine and cotinine clearance rate. Manhattan and QQ plots for the secondary analysis are presented in Additional file 1: Figure S1.

Altogether, 55 unique CpG sites in 40 genes (22 of which were novel in 22 genes) were identified in our discovery and secondary EWAS analyses.

### Pathway analyses

We performed gene-level pathway analyses for the 40 genes identified in our EWAS (discovery and secondary) and observed an enrichment of 31 specific gene networks and pathways (FDR < 0.05) (Additional file 2: Table S1). The top ones were colorectal cancer metastasis signaling, thrombin signaling, and purigenic receptor signaling. We also performed CpG-level gene ontology adjusting for non-uniform distribution of probes per gene on the methylation array (450 k) and observed no enrichment (FDR > 0.05) of biological processes (Additional file 2: Table S1).

### Genetic association analysis

To assess the potential genetic influence on cotinine levels, we tested the association between cotinine levels and 46,780 polymorphic SNPs in all highlighted genes and observed 20 SNPs (in 9 genes) significantly associated (FDR < 0.05) with cotinine levels (Additional file 3: Table S2; QQ plot in Additional file 4: Figure S2). The strongest association was observed for rs187669467 in *ARHGAP44* (FDR = 2.6 × 10^−02^) explaining 7% of variance in cotinine levels. We also tested the association of 375 genome-wide significant SNPs identified in the cotinine GWAS meta-analysis [26] in our discovery sample and observed 152 SNPs to be nominally associated (*P <* 0.05) with cotinine levels. The 20 SNPs highlighted in the current study were not genome-wide significant in the Ware et al. study [26].

### Methylation quantitative trait loci analysis

**Fig. 3:**
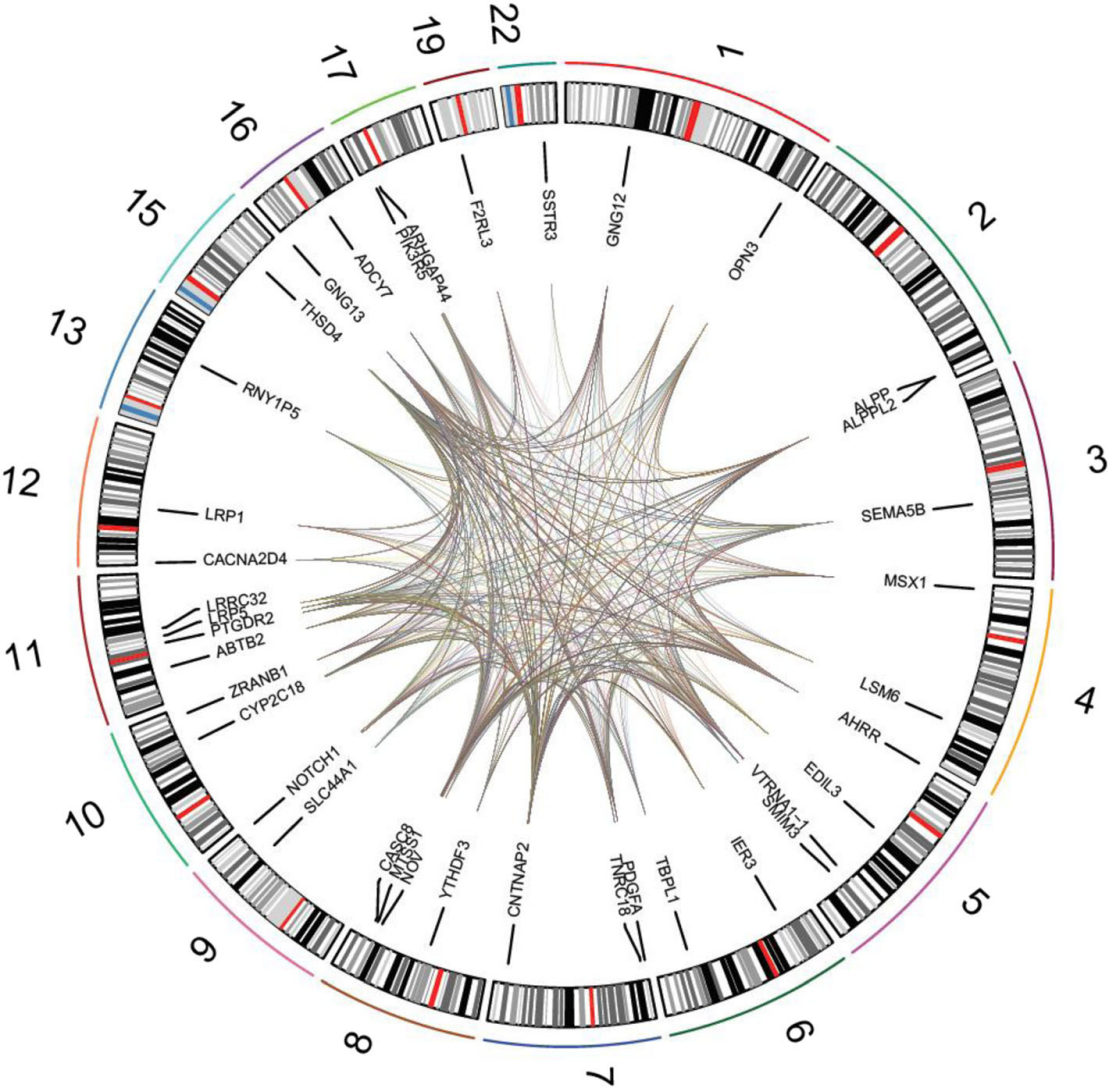
Circos plot showing the presence of methylation quantitative trait loci among the 55 highlighted CpG sites and SNPs in 40 highlighted genes

We further assessed the influence of genotypes on methylation levels of the highlighted CpG sites. We performed meQTL analysis for 46,780 polymorphic SNPs within the 40 genes (with 50 kb flanking regions) identified in our discovery and secondary EWAS and identified 124 *cis* meQTLs (Additional file 5: Table S3) and 3898 *trans* meQTLs (Additional file 6: Table S4) (FDR < 0.05). *Cis* meQTLs were observed for 22 CpG sites (average 5 SNPs per CpG, range 1–23) and 73 SNPs (average 2 CpG sites per SNP, range 1–7) in 15 genes, while *trans* meQTLs were observed for all 55 CpGs (40 genes; average 70 SNPs per CpG, range 2–198) and 321 SNPs (31 genes; average 12 CpG sites per SNP, range 1–51). Figure 3 illustrates the ample *cis* and *trans* meQTLs identified with a circos plot (Circos.ca [32]).

### Mediation analysis

At loci where genotype was associated with both methylation (meQTL) as well as cotinine levels (19 SNPs overlap), we performed mediation analysis implemented with causal inference test (CIT) to investigate the role of DNA methylation as a molecular mediator in the observed association between genotype and cotinine levels (Additional file 7: Figure S3). CIT is a widely used approach to infer causal indirect effects of a genetic variant on an outcome using a series of statistical tests (see the “Methods” section and Additional file 7: Figure S3) evaluating conditional independencies between covariates in order to distinguish a mediated effect of the genetic variant (G) on an outcome (cotinine; C) through an intermediate (methylation, M) from a reverse cause and a common cause (pleiotropic) effect [33, 34].

Mediation analysis of 19 SNPs that associated with both cotinine levels and methylation revealed a trend (*P* < 0.05) for methylation at seven CpG sites (in *LSM6*, *TNRC18*, *MTSS1*, *CASC8*, *PTGDR2*, *THSD4*, and *TMEM220-AS1*) being a molecular mediator between the effects of SNPs (in *GNG12*, *CNTNAP2*, *CYP2C18*, *ABTB2*, and *THSD4*) and cotinine levels (Additional file 8: Table S5). With a *cis* effect observed between rs532712997 and cg26589665 in *THSD4*, the remaining six CpG sites showed mediating effects with multiple SNPs in *trans*. These results indicate that alteration of methylation at these CpG sites may be a mediator in the causal pathway between genotype and observed cotinine levels, rather than a direct consequence of nicotine exposure/smoking. These seven CpG sites (Additional file 8: Table S5) bind multiple transcription factors (TF), include DNase I hypersensitive site (DHS), and overlap with enhancers (Additional file 9: Table S6 and Additional file 10: Table S7).

### Replication

To replicate our findings, we assessed the association between serum cotinine levels and methylation at the 55 CpG sites identified in our discovery and secondary EWAS in a Dutch population-based sample from the Netherlands Twin Register (NTR) as well as in an independent Finnish population cohort DIetary, Lifestyle, and Genetic determinants of Obesity and Metabolic syndrome (DILGOM). Among the 55 CpGs identified in our EWAS, 23 CpG sites were nominally associated (*P* < 0.05) with cotinine levels in NTR (*N* = 450) and 11 CpG sites in DILGOM (*N* = 79) with consistent direction of association, i.e., higher methylation associated with lower cotinine levels (Additional file 10: Table S7).

To replicate the trend observed in mediation analysis, we followed the same procedure as in FTC. For the 11 CpG sites where methylation levels were nominally associated (*P <* 0.05) with cotinine levels in the DILGOM sample (Additional file 10: Table S7), we performed genetic association and meQTL analysis. Altogether, eight SNPs were observed as meQTLs and were also nominally associated with cotinine levels (*P <* 0.05) in the DILGOM sample. We employed CIT to assess mediation between these eight SNPs and 11 CpG sites and observed a trend (*P <* 0.05) for mediation at seven CpG sites in *AHRR*, *SLC44A1*, *NOTCH1*, *F2RL3*, and *CASC8* (Additional file 11: Table S8) with cg25305703 in *CASC8* consistent with the mediation trend observed in FTC.

## Discussion

Smoking has a major influence on methylation changes across the genome, as evidenced by numerous EWAS conducted in last few years and extensively elaborated in previous reviews [1, 35]. Cotinine is a reliable measure of recent nicotine exposure compared to self-reported smoking status or smoking quantity that are prone to reporting bias [24, 25]. In this study, we assessed the association of serum cotinine level with genome-wide DNA methylation in a sample of biochemically verified current smokers (serum cotinine > 4.85 ng/ml) from the Finnish Twin Cohort. With a half-life of 15–20 h, cotinine is a reliable indicator of recent nicotine exposure [25]. In our relatively small sample (*N* = 310), we replicated several previously reported findings (33 CpG sites), many of which were originally identified in studies utilizing self-reported smoking measures in much larger samples (*N* ~ 1000 or more) [2–4], thus showing that a biomarker with biological proximity to the phenotype provides high statistical power.

Aside from the consistently reported association with CpG sites in *AHRR*, *F2RL3*, *ALPPL2*, *IER3*, and *MTSS1*, which have previously been discussed extensively in the context of smoking [3, 9, 10, 22], we identified novel loci pointing to smoking-related genes such as *THSD4*, *LSM6*, *CACNA2D4*, and *CYP2C18.* An interesting novel candidate identified in our EWAS is *THSD4* (thrombospondin type 1 domain containing 4). Genetic variants in this gene have been associated with several smoking-related phenotypes including nicotine dependence [36], smoking cessation success in clinical trials [37], and lung function affected by smoking [38]. Other genes highlighted in our EWAS with genetic variants associated with smoking-related phenotypes include *LSM6* and *CNTNAP2* associated with nicotine dependence [36] and *CACNA2D4* with pack years (indicator of lifelong accumulated smoking exposure) [39]. In our data, SNPs in *CNTNAP2* and *THSD4* genes were significantly associated with cotinine levels, in line with these previous findings. Some of the pathways highlighted among the genes identified in our EWAS have been implicated in smoking-associated aberration in cancer pathogenesis [40, 41] and pulmonary disorders [42–44], highlighting the clinical significance of our findings.

The level of cotinine is influenced not only by intake (such as smoking pattern and smoking quantity) but also by the rate of clearance of cotinine [28]. Therefore, we utilized a GRS for NMR (a proxy for nicotine and cotinine clearance rate) to account for the impact of this in our secondary analysis and identified five additional novel loci but also observed that half of the associations from our discovery EWAS were no longer significant. A plausible explanation for this observation could be that adding the GRS adjusts for the genetic variation of nicotine metabolism rate (normal vs faster metabolizers) among the individuals analyzed. Associations which disappear when the NMR GRS is added to the model may be related to the impact of nicotine metabolism rate on nicotine intake or nicotine/cotinine metabolism. Among such associations (no longer genome-wide significant after adjusting for the NMR GRS) are CpG sites in genes *AHRR* and *CYP2C18*, members of the metabolic pathways involved in nicotine and bupropion (prescription medication for smoking cessation [45]) degradation and xenobiotic metabolism [46]*. CYP2C18*, a member of the cytochrome P450 (CYP) family, and *AHRR*, a regulator of CYP genes, are both involved in xenobiotic (which includes components of cigarette smoke) metabolism. Altered expression of *CYP2C18* in the context of smoking has been reported previously [47]; however, the role of methylation as a plausible regulator in such observed alterations warrants further experimental investigation. It should be noted that although just three SNPs used in the NMR GRS account for a relatively high proportion (~ 30%) of variance in NMR, a large portion of variance remains unaccounted for and this is a major limitation of our study. The three independent SNPs constituting the GRS were identified in the Finnish population sample and may not capture the same effects across other populations (as we observed with the NTR replication sample).

As some of the CpGs identified in our EWAS were annotated to genes that have previously been linked to smoking-related phenotypes (36–39), we examined the role of genetic variants in the genes identified in our EWAS. It is noteworthy that a plethora of associations between the methylation levels of highlighted CpG sites and SNPs were observed in both *cis* and *trans.* We can speculate a cross-genome interplay between nicotine exposure-associated DNA methylation and genetic variation in the highlighted genes [48, 49]. Some of the *cis* meQTLs also act in *trans* with other CpG sites. Most of the CpG sites bind multiple TFs, overlapping enhancers or DHS sites hinting at global-directed networks at play [48, 50]; however, these may be independent effects occurring simultaneously. It should also be noted that we tested meQTLs only among genes highlighted in our analysis potentially missing other *cis* and *trans* meQTLs in the whole genome. Interestingly, for the 19 meQTLs that were also associated with cotinine levels, we observed a trend of molecular mediation by methylation at seven CpG sites. These results suggest that alterations in methylation of these nicotine exposure-associated CpG sites might not always be a consequence of smoking as commonly suggested, but rather methylation at such CpG sites may act as a causal mediator (molecular mechanism) in regulating the effect of genetic variation (in genes that modulate nicotine intake and/or nicotine and cotinine metabolism rate) on cotinine levels or smoking behavior. We observed support for these findings in an independent replication sample (DILGOM). These findings are particularly interesting as these SNPs do not overlap SNPs in the probes. We observed *cis* mediation between CpG sites and SNPs in *THSD4* (FTC) and *AHRR* (DILGOM) both involved in smoking [36–38, 51], while *trans* mediation in CpG sites that bind multiple TFs or overlap DHS sites and enhancers providing plausible action mechanism for molecular mediation. These findings are crucial and provide caution to interpretation of smoking-related EWAS but require further experimental evidence.

Cotinine, even though a reliable measure of nicotine exposure, is not specific to tobacco smoking. Other potential sources of nicotine, such as smokeless tobacco (e.g., snus), e-cigarettes, and/or nicotine replacement therapy, could also contribute to cotinine levels; however, the likelihood of such alternative sources in our sample was low (see the “Methods” section). There have been only a few EWAS so far using cotinine as a continuous phenotype [51, 52], both of which were performed in samples including smokers and non-smokers. When considering replication of prior findings, it should be noted that all the previous EWAS we used to assess the novelty of our findings (see “Methods”) were conducted in samples including both smokers and non-smokers. We cannot rule out that our novel findings might be specific to current or heavy smoking as we limited our analyses to self-reported current smokers with elevated cotinine levels only. Having included only current smokers, the range of variability is limited (in contrast to studies including both smokers and non-smokers) resulting in smaller effect sizes observed. Similar results (small effects) were noted by Zhang et al. [52] and Su et al. [22] when restricting analysis to current smokers only. Although we observed a great overlap with previous findings, non-overlapping associations could be due to differences in study design and sample (smokers and non-smokers in previous publications versus current smokers only in our study), population specificity of methylation levels [12], and precision of cotinine measurement technique (mass spectrometry versus immunoassay) [53]. The lack of replication of the novel associations identified in our EWAS in the NTR and DILGOM samples may be due to several aforementioned factors including population specificity, differences in precision of cotinine measurement (mass spectrometry (FTC and DILGOM) and immunoassay (NTR)) and small sample size (DILGOM *N* = 79), or small and inconsistent effects of novel associations. Even though we replicated several prior associations between smoking and methylation, the novel associations identified in our study, while biologically meaningful, would require replication in other larger samples.

## Conclusions

In conclusion, we examined the epigenetic signature of nicotine exposure in a sample of biochemically verified current smokers and identified several novel loci, including genes involved in nicotine degradation and metabolism. Our study is the first cotinine EWAS in which the rate of nicotine clearance is accounted for, allowing us to discover smoking-pertinent novel associations which may be specific to regular/heavy smoking. We further expose genetic influences on methylation and provide suggestive evidence for the role of methylation as a molecular mediator between genetic variation and cotinine levels, as opposed to a direct consequence of nicotine exposure in some of the genes.

## Methods

### Study sample

The study sample of cotinine verified current smokers comes from the Finnish Twin Cohort (FTC) [54], a population-based longitudinal study designed to examine genetic and environmental determinants of health-related behaviors. A total of 310 individuals (51 full monozygotic pairs, 44 full dizygotic pairs, and 120 twin individuals without a co-twin) were included in our discovery analysis. Genome-wide DNA methylation was assessed with Infinium HumanMethylation450 BeadChip in peripheral blood using standard protocols [55]. The average age in the sample was 29.5 years (SD 14.2, range 21–69), and 52% were females. Genotype data imputed to 1000Genomes phase 3 (processing and imputation of the genotype data has been described in detail previously [31]) was available for 304 of these individuals, which were consequently included in the secondary analysis.

### Phenotype data

Current smokers were selected based on the threshold of serum cotinine above 4.85 ng/ml, as suggested by Benowitz et al. [56]. Selecting current smokers with a threshold of cotinine > 4.85 ng/ml instead of 10 ng/ml was done to maximize sample size available for discovery analysis, and sensitivity analysis (results not shown) was performed to ensure that using a higher threshold did not affect the results and subsequent inference. Cotinine was measured from frozen serum samples using high-precision LC-MS/MS at University of Toronto, Canada (*N* = 242) [57] and at the Metabolomics Unit, Institute for Molecular Medicine Finland (FIMM), University of Helsinki, Finland (*N* = 68). The protocols and instrument parameters have been described previously [31, 58, 59]. Average cotinine in our discovery sample (*N* = 310) was 192.7 ng/ml (median = 178.8, SD = 148.4, range = 5.1–820.5). In our discovery sample, two individuals (with cotinine levels above 100 ng/ml) reported using nicotine replacement therapy and none declared use of smokeless tobacco. E-cigarette use was non-existent in Finland at the time of data collection.

### Replication samples

#### DILGOM

To replicate our findings, we utilized a Finnish population cohort DILGOM (DIetary, Lifestyle, and Genetic determinants of Obesity and Metabolic syndrome) [60, 61]. Briefly, DILGOM originates from the population-based national FINRISK 2007 study and includes a total of 631 unrelated Finnish individuals aged 25–74 years from the Helsinki area. A total of *N* = 79 individuals with methylation data from the peripheral blood, genome-wide genotype data imputed to 1000Genome phase I, and serum cotinine > 4.85 ng/ml (current smokers) were analyzed. The average age of the sample was 51 years (SD 13.5, range 25–72), and 44% were females. Cotinine was measured with gas chromatograph mass spectrometry at the Laboratory of Analytical Biochemistry at the Institute of Health and Welfare, Helsinki, Finland, as described earlier [57]. All 79 individuals were self-reported current smokers. Average cotinine levels were 134.2 ng/ml (median = 129.6, SD = 98.0, range 5.0–408.8).

#### Netherlands twin registry

The subjects participated in longitudinal survey studies from the Netherlands Twin Register (NTR) [62] and in the NTR biobank project [63]. Data from 450 samples from 446 individuals (58% females) were analyzed. For four individuals, two longitudinal blood samples were included. The blood sampling procedure [63], Illumina 450 k methylation data [64], and nicotine measurements have been described in detail previously [65]. A standard threshold of plasma cotinine> 50 ng/ml (for immunoassay measurements) was used to select current smokers [66]. Average cotinine levels were 309.6 ng/ml (median = 229.0, SD = 300.5, range = 50.0–2329.0).

#### DNA methylation data processing and analysis

DNA methylation data was preprocessed and normalized using the pipeline suggested by Lehne et al. [67] implemented using R packages “minfi” [68] and “limma” [69]. We modified the pipeline to accommodate the relatedness in our sample. Altogether the quality control involved exclusion of probes with detection *P* > 1 × 10^− 16^, bead count < 3 (estimated with “wateRmelon” R package [70]) and call rate < 98%. Samples with sample call rate < 98% and sex mismatch were further excluded. After quality control, 418,302 probes and *N* = 310 samples remained (FTC data). Quantile normalization stratified on probe type, color channel, and probe subtypes was performed to obtain a methylation beta matrix. The first 30 principal components (PCs) from the control probes were calculated to adjust for technical bias. White blood cell subtypes (CD8T, CD4T, Natural Killer cells, B cell, monocyte, and granulocyte) were estimated using “FlowSorted.Blood.450 k” [71] within the “minfi” R package based on a modified version of the Houseman algorithm [72] and included in the regression model. Intermediary residuals were estimated by regressing the methylation beta values on age, sex, body mass index (BMI), 30 control probe PCs, and white blood cell types. PCs from these intermediary residuals were further included in the final regression model to adjust for any unaccounted global covariation. To analyze the association between methylation (dependent variable) at each CpG site and serum cotinine levels (explanatory variable), we used linear mixed effects model with “lmer” function implemented in “lme4” R package [73]. Age, sex, BMI, batch variable (site of cotinine measurement), 30 control probe PCs, and 10 intermediary residual PCs were included as covariates (fixed effects), while family identifier and zygosity were included as random effects to account for the relatedness in our sample. To avoid spurious associations, we further applied ad hoc exclusion of probes reported by Zhou et al. [74] to affect beta methylation at the CpG (probes with non-unique mapping, inconsistent extension base, SNP in the extension base, and overlap with any known SNPs with global minor allele frequency (MAF) and MAF in a Finnish population > 1%).

We applied false discovery rate (FDR) adjustment to the *p* values for multiple testing correction and considered FDR-adjusted *P* < 0.05 as statistically significant. Manhattan and QQ plots were produced using the R package “qqman” [75]. Lambda value estimates for the QQ plot were calculated using the “estlambda” function in R package “GenABEL” [76]. For replication of our results, analysis pipeline was identical for the replication sample NTR. For the DILGOM sample, analysis was identical except that instead of a mixed effects model, a linear model was used as all individuals were unrelated.

#### Secondary analysis

To account for the individual rate of nicotine metabolism, we included the GRS for NMR in the regression model (described above) as an additional covariate. We extracted the genotypes for the three independent SNPs (rs56113850, rs113288603, and rs12461964) and calculated the weighted mean of minor allele counts to calculate the GRS, as described previously [31]. One of the top variants (indel esv2663194) identified in the NMR GWAS meta-analysis [31] was not available in our samples (discovery and replication). GRS calculation and analysis pipeline was identical for the replication samples.

#### Literature search

We used the same literature search strategy as Joehanes et al. [2]. Briefly, PubMed literature database was queried using medical subject heading (MeSH) terms of DNA methylation and smoking. To limit duplication of efforts, we used the supplementary data on literature search used by Joehanes et al. which included articles until 2015 and applied additional filter on year (starting 2015) along with a filter for species (human). We reviewed the abstracts to determine if the studies were as follows: (1) performed in healthy adult human populations (also excluding maternal smoking), (2) sample type analyzed was peripheral blood, (3) studies assessed genome-wide methylation only, and (4) public reporting of *P* values and gene annotations for the CpGs identified was available. A total of 24 publications met all the inclusion criteria and are listed in the Additional file 12: Table S9. A compiled list of CpGs (19,208 CpG sites) and associated gene names (8582 genes) from all 24 studies were utilized to assess novelty of our findings.

#### Annotation and pathway analysis

All statistically significant CpG sites were annotated using the data aggregated by Zhou et al. [74]. For the probes that did not have a gene name listed, the name of the nearest gene was fetched from Ensembl [77]. This gene list was analyzed with the Ingenuity Pathway Analysis software (IPA; Ingenuity® Systems, https://www.qiagenbioinformatics.com/) to identify gene networks at play. We applied multiple testing correction and an enrichment for genes functioning in signaling and metabolic pathways [78] was considered significant when FDR < 0.05. A total of 38 of the 40 highlighted genes were recognized by IPA. We also performed pathway analysis (Gene Ontology) to assess enrichment of biological processes among the top 55 CpG sites while taking into account the non-uniform CpG probe distribution per gene [79] on the 450 k array with R package “missMethyl” [80] and corrected for multiple testing and considered FDR *< 0.05* significant.

#### Association of cotinine with genetic variants

As the genes identified in our EWAS harbor genetic variants associated with smoking behavior phenotypes, such as nicotine dependence [36], we examined whether SNPs in the genes identified in our EWAS analysis associate with cotinine levels. We performed association analysis using mixed effects model implemented as “lmer” function in “lme4” R package. Cotinine levels were treated as dependent variable, while genotype dosage (coded as 0, 1 or 2 for copies of effect allele), age, sex, and BMI were included as fixed effects in the model. Family and zygosity were included as random effects to account for the relatedness in our sample. Only polymorphic SNPs (*N* = 46,780) were tested for association with cotinine levels. Analysis was otherwise identical for the DILGOM sample except that linear model was used instead of mixed effects model, as all individuals were unrelated. To estimate proportion of variance explained by individual SNPs, we subtracted the R^2^ of the model including only covariates (age, sex, BMI) from the R^2^ of the model also including the SNP.

#### Methylation quantitative trait loci analysis

To examine the association between methylation and SNPs within the 40 genes (with 50 kb flanking regions; gene boundaries are available in Additional file 13: Table S10), we used R package “MatrixEQTL” [81]. Only polymorphic SNPs (*N* = 46,780) were tested using linear model setting with a *cis* distance of 2.5 Mb. The longest gene was approximately 2.4 Mb (Additional file 13: Table S10); thus, a *cis* distance of 2.5 Mb was chosen to ensure that all possible combinations of SNPs, and the CpG within a gene are tested. All association tests between remaining SNP and CpGs were considered *trans*. Genotypes were coded as copies of effect allele (0, 1, or 2) and methylation data was extracted for the 55 probes from the CPACOR normalized data set used in the EWAS. We included, age, sex, BMI, white blood cell counts, control probe PCs, and cotinine as covariates in the model. In addition, owing to the relatedness in our sample, we included a covariance matrix based on CpG sites being tested in the model. Results of meQTL analysis were visualized using R package “RCircos” [82].

#### Mediation analysis

For the SNPs that were significantly associated with both cotinine levels and methylation, we investigated the potential of methylation (M) being a mediator between the effects of genetic variant (G) on outcome cotinine levels (C) using causal inference test (CIT) [83]. In CIT, four conditions are tested to assess consistent causal mediation as described by Millstein et al. [83]. Conditions for CIT are (1) G is associated with C, (2) G is associated with M conditional on C, (3) M is associated with C conditional on G, and (4) G is independent of C conditional on M. We implemented CIT analysis with R package “cit” and function “cit.cp” with 50 permutations for conditional analysis and included, age, sex, BMI, family, and zygosity as covariates. We report the overall *p* value (omnibus *p* value) based on collective conditional analysis and considered *P* < 0.05 as nominally significant.

## Additional files

Additional file 1: **Figure S1.** Manhattan and QQ plots showing epigenome-wide association results from secondary analysis when accounting for the rate of nicotine metabolism using a GRS. (A) QQ plot showing observed versus expected − log_10_(*P*) for association at all loci. (B) Manhattan plot showing chromosomal locations of − log_10_(*P*) for association at each locus. All CpG sites with FDR < 0.05 are highlighted in green and the top gene for each of the highlighted loci is labeled. (PDF 171 kb)

Additional file 2: **Table S1.** Gene network (IPA) and pathway analysis (Gene Ontology) of EWAS results. (XLS 3125 kb)

Additional file 3: **Table S2.** SNPs in the 40 top genes significantly associated (FDR *p* < 0.05) with cotinine levels. (XLS 14 kb)

Additional file 4: **Figure S2.** QQ plot for genetic association analysis of cotinine levels and 46,780 SNPs in 40 genes. (PDF 17 kb)

Additional file 5: **Table S3.** *Cis* acting methylation quantitative trait loci in the 40 genes highlighted in the EWAS. (XLS 31 kb)

Additional file 6: **Table S4.** *Trans *acting methylation quantitative trait loci in the 40 genes highlighted in the EWAS. (XLS 687 kb)

Additional file 7: **Figure S3.** Mediation analysis to assess whether DNA methylation is a causal mediator to the observed association between genetic variants and cotinine levels. (PDF 588 kb)

Additional file 8: **Table S5.** Mediation analysis omnibus *P* values for the 19 meQTLs associated with serum cotinine levels. (XLS 33 kb)

Additional file 9: **Table S6.** Transcription factor binding site information for 55 highlighted CpG sites based on ENCODE data. (XLS 96 kb)

Additional file 10: **Table S7.** CpG sites showing significant (FDR *p* < 0.05) association with cotinine levels in regular smokers in FTC and replication results for top 55 CpG sites in NTR and DILGOM. (XLS 42 kb)

Additional file 11: **Table S8.** Results for CIT performed on DILGOM sample replicated CpG sites and SNPs associated with their methylation levels as well as cotinine levels. (XLS 10 kb)

Additional file 12: **Table S9.** Literature search results for smoking EWAS. (XLS 9 kb)

Additional file 13: **Table S10.** Gene boundaries, based on Ensembl transcripts for the 40 genes highlighted in the EWAS. (XLS 10 kb)

## Abbreviations

BMI: Body mass index
CIT: Causal inference test
DHS: DNase I hypersensitive site
DILGOM: DIetary, Lifestyle, and Genetic determinants of Obesity and Metabolic syndrome
EWAS: Epigenome-wide association study
FDR: False discovery rate
FTC: Finnish Twin cohort
GRS: Genetic risk score
GWAS: Genome-wide association study
IPA: Ingenuity pathway analysis
LC-MS/MS: Liquid Chromatography with tandem mass spectrometry
MAF: Minor allele frequency
meQTL: Methylation quantitative trait loci
MeSH: Medical subject heading
NMR: Nicotine metabolism ratio
NTR: Netherlands Twin Register
PC: Principal components
QQ: Quantile-quantile
SNP: Single nucleotide polymorphism
TF: Transcription factor
