# Supplementary figures and images for: Epigenome-wide association study of serum cotinine in current smokers reveals novel genetically driven loci
Source: Clin Epigenetics. 2019 Jan 5;11:1. doi: 10.1186/s13148-018-0606-9 (PMC6321663; doi:10.1186/s13148-018-0606-9)

A)

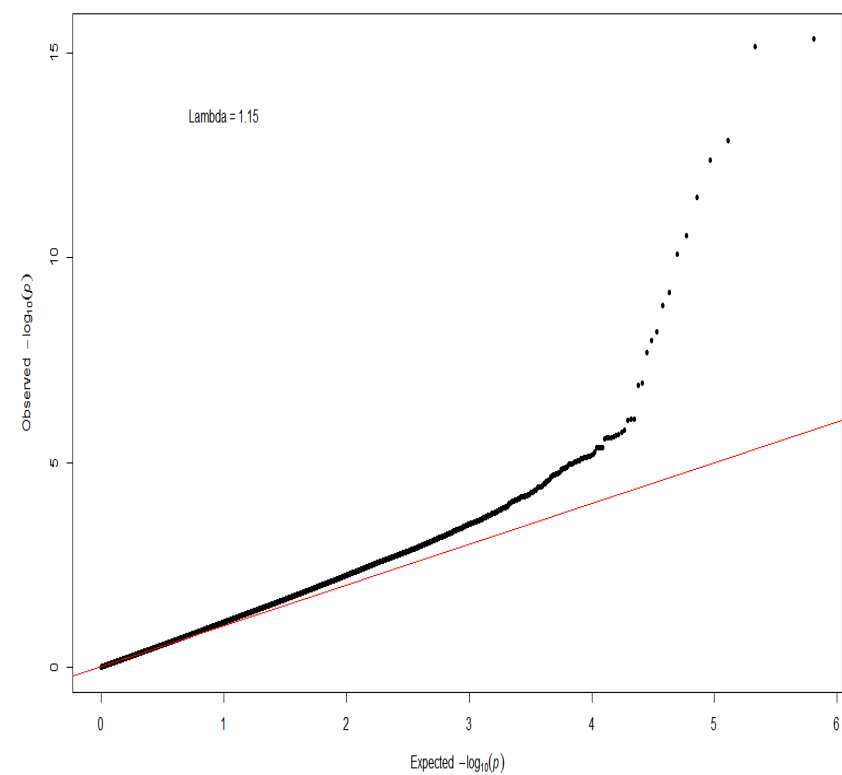

B)

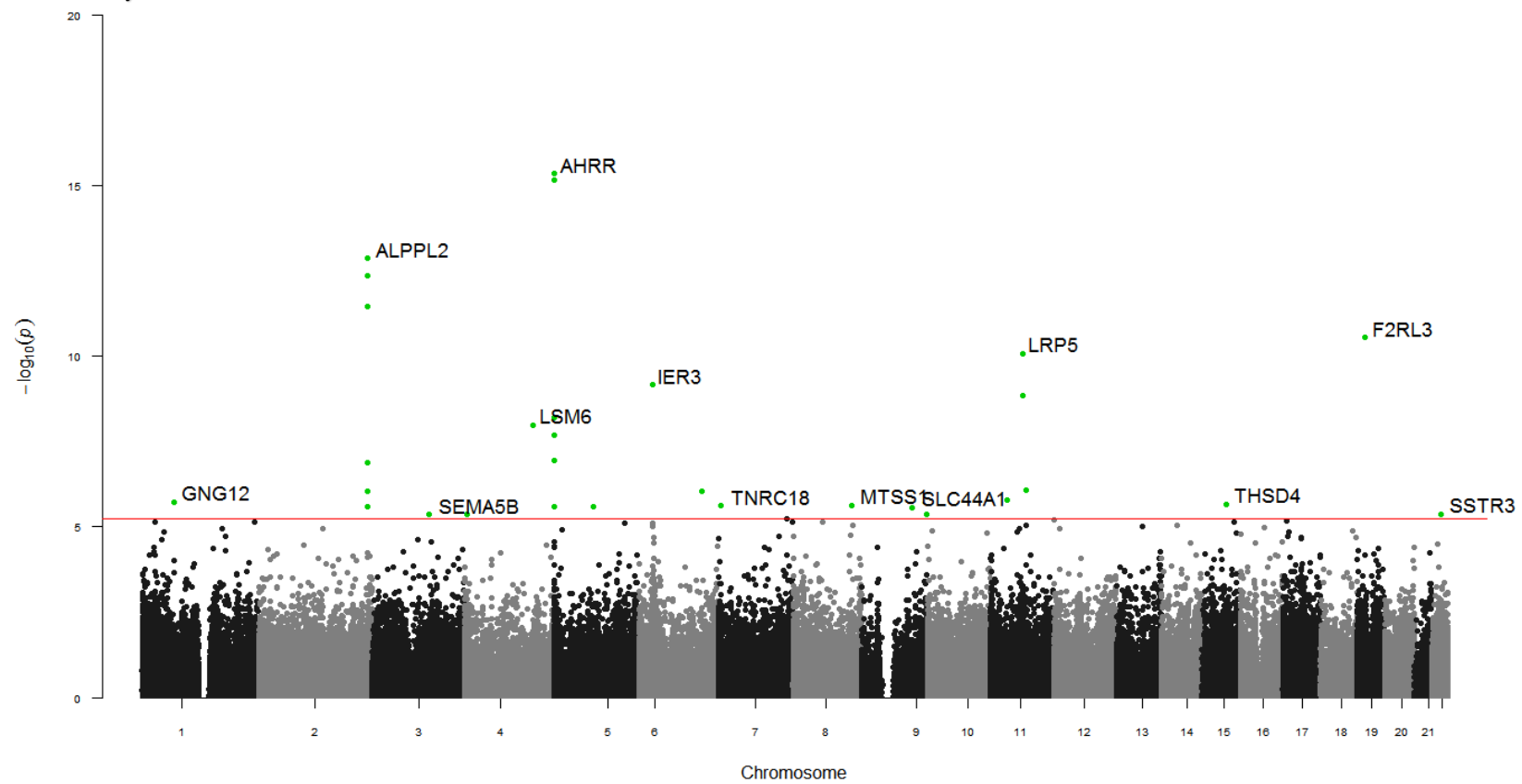

Supplement: Supplementary file 1 — Figure S1. Manhattan and QQ plots showing epigenome-wide association results from secondary analysis when accounting for the rate of nicotine metabolism using a GRS. (A) QQ plot showing observed versus expected − log10(P) for association at all loci. (B) Manhattan plot showing chromosomal locations of − log10(P) for association at each locus. All CpG sites with FDR < 0.05 are highlighted in green and the top gene for each of the highlighted loci is labeled. (PDF 171 kb) [file 13148_2018_606_MOESM1_ESM.pdf]

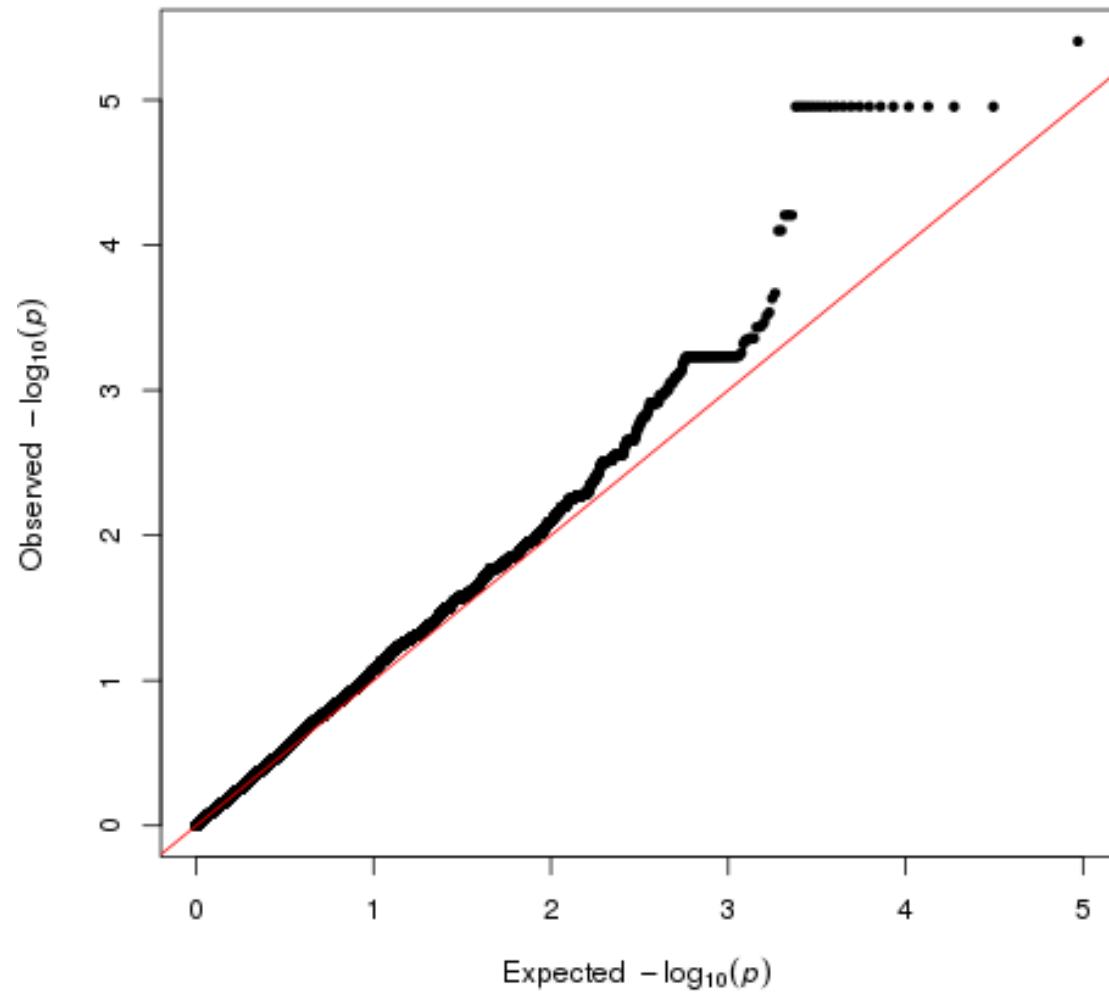

Supplement: Supplementary file 4 — Figure S2. QQ plot for genetic association analysis of cotinine levels and 46,780 SNPs in 40 genes. (PDF 17 kb) [file 13148_2018_606_MOESM4_ESM.pdf]
