# Supplementary material for: Epigenome-wide association study of serum cotinine in current smokers reveals novel genetically driven loci
Source: Clin Epigenetics. 2019 Jan 5;11:1. doi: 10.1186/s13148-018-0606-9 (PMC6321663; doi:10.1186/s13148-018-0606-9)
Supplement: Supplementary file 7 — Figure S3. Mediation analysis to assess whether DNA methylation is a causal mediator to the observed association between genetic variants and cotinine levels. (PDF 588 kb) [file 13148_2018_606_MOESM7_ESM.pdf]

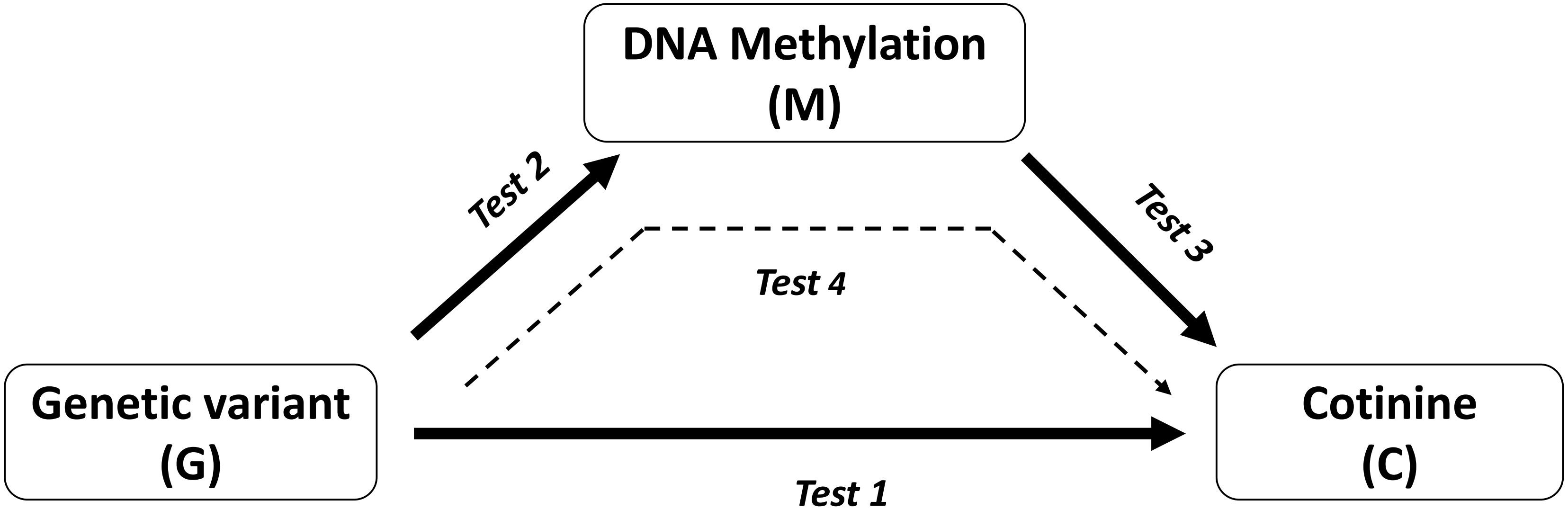

Four Component test of CIT: assess whether genetic variant (G) lead to variation in a quantitative trait (*C,* Cotinine) through changes in DNA methylation (M).

Test 1: G is associated with C
Test 2: G is associated with M conditional on C
Test 3: M is associated with C conditional on G
Test 4: G is independent of C conditioned on M
